# Improved blood tests for cancer screening: general or specific?

**DOI:** 10.1186/1471-2407-11-499

**Published:** 2011-11-30

**Authors:** Ian A Cree

**Affiliations:** 1 Translational Oncology Research Centre, Queen Alexandra Hospital, Southwick Hill, Portsmouth, PO6 3LY, UK

## Abstract

Diagnosis of cancer at an early stage leads to improved survival. However, most current blood tests detect single biomarkers that are of limited suitability for screening, and existing screening programmes look only for cancers of one particular type. A new approach is needed. Recent developments suggest the possibility of blood-based screening for multiple tumour types. It may be feasible to develop a high-sensitivity general screen for cancer using multiple proteins and nucleic acids present in the blood of cancer patients, based on the biological characteristics of cancer. Positive samples in the general screen would be submitted automatically for secondary screening using tests to help define the likelihood of cancer and provide some indication of its type. Only those at high risk would be referred for further clinical assessment to permit early treatment and mitigate potential overdiagnosis. While the assays required for each step exist, they have not been used in this way. Recent experience of screening for breast, cervical and ovarian cancers suggest that there is likely to be widespread acceptance of such a strategy.

## Introduction

Several successful screening programmes are already well established, but these are currently applicable only to common cancers such as the faecal occult blood test [1] for colorectal cancer, mammography for breast cancer [2] and, of course, cervical cytology for cervical cancer and dysplasia, which is becoming ever more sophisticated [3,4]. Despite many attempts, blood tests have a less distinguished record. For instance, prostate-specific antigen screening is widely used despite its well-publicised problems [5]. It remains controversial and generates large numbers of papers every year (2, 032 were indexed in PubMed through 2010 using the search terms 'screening', 'prostate specific antigen' and 'cancer'). Many other tumour markers have been described, usually in relatively small studies, and few make it through to clinical use. Cancer antigen 125 (CA 125) was first described as a marker of ovarian cancer in 1981 [6] and is still being evaluated as a potential screening test [7,8].

**Figure 1:**
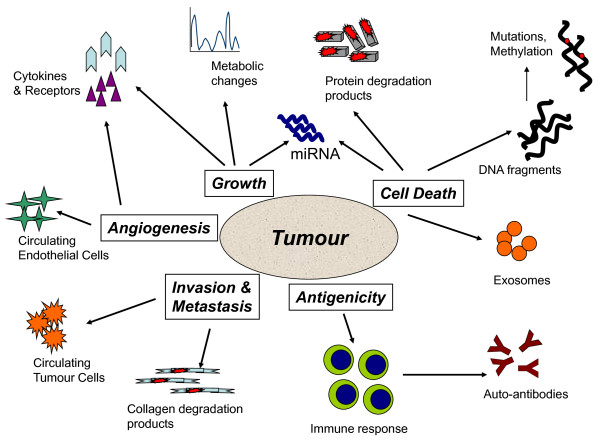
**Tumour biomarkers in blood reflect the major processes resulting in tumour formation by cancer cells and the host reaction**.

Despite its history, blood-based screening for cancer remains attractive, as it could provide inexpensive testing that would arguably be more acceptable to patients and easily incorporated into an annual checkup, which might include cholesterol and other assays of general health. This idea was judged too risky to be funded when put forward in 2005, but six years later, the recent review along similar lines by Hanash *et al*. [9] has shown how fast the necessary underlying science is advancing. There is no doubt that cancers have characteristics that could be detected by performing blood-based screening tests (Figure 1). In 2000, Hanahan and Weinberg [10] published their seminal paper describing the Hallmarks of Cancer, and many authors since then have described changes in blood related to these characteristics. Hanahan and Weinberg pointed out that cancer cell growth is the result of self-sufficiency in growth signals and insensitivity to antigrowth signals. Such signals are often mediated by growth factors, which may rise above normal levels in peripheral blood [11]. Growth has consequences: Even in the early stages there may be detectable metabolic changes [12,13], though these often lack specificity [14]. Cancer cells also upregulate mechanisms that allow them to evade apoptosis, and some of these also cause the release of cytokines into blood [15,16]. Many tumours also have increased cell turnover: They grow because they divide faster than they die, but there is still increased cell death by apoptosis or necrosis. This overloads the local clearance mechanisms for dead cells in tissues and leads to the appearance of partly caspase-digested proteins and DNA fragments in peripheral blood [17-19]. The amount of DNA present is increased and may contain mutations or altered methylation [20-23]. More recently, the presence of RNA, particularly in the form of miRNA [24,25] and exosomes [25-27] derived from cancer cells in blood, has opened up new avenues of research. Tumours require the ability to make new blood vessels, and many therefore produce proangiogenic factors, which can be found in blood [28,29]. They also increase the number of endothelial cell precursors in blood [29]. Immunological abnormalities are common in many cancer patients, with the appearance of autoantibodies to p53 and other intracellular antigens [30]. Finally, malignant cells invade and metastasise. While few metastatic cells survive and grow, their presence can be detected in blood by using sensitive assays [31].

## Discussion

The question, therefore, is, Is it possible to use a general screen that might select a set of individuals from the general population who could then be screened further, initially by using the same blood sample, to confirm whether they have cancer and give some guidance as to type? While the objective of general population screening is to identify individuals with a high risk of cancer, the aims of secondary and finally diagnostic screening would be, respectively, to (1) confirm positivity and (2) determine the type of cancer to assist in the choice of further investigations to perform in a selected at-risk group. The advantage of the two-stage method proposed is that those tests showing greatest specificity often have limited sensitivity. Circulating free DNA is a good example of a test with considerable sensitivity [32,33], but measurement of this alone in patients with inflammatory or other conditions could lead to an unacceptably high false-positive rate and might give little indication of the site of the tumour [34,35]. Equally true is that studies of gene mutations or autoantibodies may have greater specificity but lower sensitivity [21,30].

Preventive maintenance is routine for any complex mechanical device and is increasingly acceptable to healthy people. In most European countries, general medical checkups are encouraged to ensure blood pressure control, and blood tests are used to guide the use of lipid-lowering drugs [36]. Highly successful screening methods are used for specific cancers, as discussed above. However, other common cancers for which no effective screening methods exist include cancers of the lung, stomach, oesophagus, pancreas, liver, head and neck, and kidney. Further, about 25% of cancer deaths occur as a result of cancers outside the 'top 10' common cancer types (Cancer Research UK: Cancer Mortality: UK Statistics. Available at http://info.cancerresearchuk.org/cancerstats/mortality/). Although the economic implications require careful study, it is possible that such screening tests could be cost-neutral to health care providers, since it is likely to be very much more expensive to treat a small number of patients for advanced cancer than to screen and treat a larger number of individuals with early cancer or precancerous conditions. The benefit to patients would be that a frequent, simple, low-risk and relatively painless investigation could prevent serious or life-threatening disease.

If the development of this strategy is successful, we will see a general change from self-referral for cancer symptoms, when treatment is often difficult, costly and unsuccessful, to regular screening using a simple blood test, permitting the treatment of small, localised tumours. New, less invasive radiological and treatment strategies are required, but these are already being introduced, such as laparoscopic surgery for colorectal carcinoma [37] and endomucosal resection of oesophageal cancers [38]. Some of the patients undergoing screening are likely to have anxieties related to the outcomes of their yearly tests, but these are arguably balanced by the knowledge that many cancers caught early are in most cases unlikely to be fatal. The excellent take-up of existing screening procedures (some quite unpleasant for patients) suggests that this is not a major issue, though it is certainly a research need and the introduction of screening procedures requires careful evaluation [39].

The major risk to patients is overdiagnosis, which has been highlighted by other screening programmes [40]. For quantitative blood tests, mitigation of this risk may be feasible by setting test thresholds appropriately so that only those at high risk are referred for further investigation, but this does require careful monitoring and quality assurance is essential.

Many of the problems alluded to are common to most translational research. Test development is the first stage (Table 1). It is unlikely that any one analyte will provide the answer, but several of them tested together or sequentially in the same sample could provide the degree of accuracy needed. This would be particularly attractive if the same or similar technologies were used for detection. The recent use of human epididymis protein 4 and CA 125 detection for ovarian cancer (now approved by the US Food and Drug Administration for monitoring disease) is a case in point where the use of multiple analytes measured by ELISA was found to provide better information than previous screening tests [41]. At present, there are numerous published studies in these areas that could be described as developmental (stage 1) or early clinical testing (stage 2), but few of these (even those with strongly positive results) go on to validation studies (stage 3), which require larger series of well-documented patients. Most stage 2 studies have too few patients to draw firm conclusions. This has recently been noted with regard to pharmacogenomic studies [42] and is by no means the preserve of cancer, where the research community at least has access to valuable information derived from large clinical trials that can be used for cancer screening. Implementation studies that examine the impact and cost-effectiveness of blood screens for cancer are very rare, mainly because they are large, complex, time-consuming and expensive to run.

**Table 1:** Stages of translation from diagnostic to clinic for diagnostic devices^a^

Stage	Stage description
Stage 1	Development of test using clinical samples
Stage 2	Early clinical testing of efficacy (sensitivity, specificity, NPV, PPV, AUROC)
Stage 3	Validation (larger numbers, defined by confidence intervals on AUROC)
Stage 4	Implementation and impact (trials or modelling to answer effectiveness questions)

^a^AUROC: area under the receiver operating characteristic curve, NPV: negative predictive value, PPV, positive predictive value.

Last, at each stage, the dissemination of results is essential. A quick search of PubMed using the terms 'early', 'detection', 'cancer' and 'validation' produced 481 articles, 100 of which were classified as reviews and only 178 of which were available as free full-text articles. *BMC Cancer *is, of course, a free full-text journal, and our experience is that publication in this format aids in the dissemination of results beyond the well-funded libraries at major universities and hospitals.

## Conclusion

General screens for cancer may be feasible but are unlikely to grow out of existing specialist screening programmes, which concentrate on particular cancer types. Multiplex approaches are likely to be most effective and, with appropriate translational support, could be practicable. There are a number of risks, mainly of overdiagnosis, which need careful management. Implementation depends on dissemination of research, and open access journals have their role to play if this is to be become a reality.

## Abbreviations

CA 125: cancer antigen 125; ELISA: enzyme-linked immunosorbent assay; miRNA: microRNA.

## Competing interests

IAC is a section editor of *BMC Cancer *and is a director of NALIA Systems Ltd, a university spinout company developing an antibody array technology for clinical use.

## Pre-publication history

The pre-publication history for this paper can be accessed here:

http://www.biomedcentral.com/1471-2407/11/499/prepub
